# The development and prevalidation of an *in vitro* mutagenicity assay based on MutaMouse primary hepatocytes, Part II: Assay performance for the identification of mutagenic chemicals

**DOI:** 10.1002/em.22277

**Published:** 2019-02-25

**Authors:** Julie A. Cox, Edwin P. Zwart, Mirjam Luijten, Paul A. White

**Affiliations:** ^1^ Environmental Health Science and Research Bureau Health Canada Ottawa Ontario Canada; ^2^ Department of Biology University of Ottawa Ottawa Ontario Canada; ^3^ Centre for Health Protection National Institute for Public Health and the Environment (RIVM) Bilthoven The Netherlands

**Keywords:** transgenic rodent, liver, genetic toxicology, regulatory toxicology, chemical mutagens

## Abstract

As demonstrated in Part I, cultured MutaMouse primary hepatocytes (PHs) are suitable cells for use in an *in vitro* gene mutation assay due to their metabolic competence, their “normal” phenotype, and the presence of the MutaMouse transgene for reliable mutation scoring. The performance of these cells in an *in vitro* gene mutation assay is evaluated in this study, Part II. A panel of 13 mutagenic and nonmutagenic compounds was selected to investigate the performance of the MutaMouse PH *in vitro* gene mutation assay. The nine mutagens represent a range of classes of chemicals and include mutagens that are both direct‐acting and requiring metabolic activation. All the mutagens tested, except for ICR 191, elicited significant, concentration‐dependent increases in mutant frequency (MF) ranging from 2.6‐ to 14.4‐fold over the control. None of the four nonmutagens, including two misleading, or “false,” positives (i.e., tertiary butylhydroquinone [TBHQ] and eugenol), yielded any significant increases in MF. The benchmark dose covariate approach facilitated ranking of the positive chemicals from most (i.e., 3‐nitrobenzanthrone [3‐NBA], benzo[*a*]pyrene [BaP], and aflatoxin B_1_ [AFB1]) to least (i.e., *N*‐ethyl‐*N*‐nitrosourea [ENU]) potent. Overall, the results of this preliminary validation study suggest that this assay may serve as a complimentary tool alongside the standard genotoxicity test battery. This study, alongside Part I, illustrates the promise of MutaMouse PHs for use in an *in vitro* gene mutation assay, particularly for chemicals requiring metabolic activation. Environ. Mol. Mutagen. 60:348–360, 2019. © 2019 The Authors. *Environmental and Molecular Mutagenesis* published by Wiley Periodicals, Inc. on behalf of Environmental Mutagen Society.

## INTRODUCTION

Regulatory evaluations of new and existing substances always require genetic toxicity assessment, and this generally includes *in vitro* assessments of mutagenic activity. Although bacterial mutagenicity testing (e.g., *Salmonella* reverse mutation test) is most commonly used for *in vitro* mutagenicity assessment, tiered testing regimes employed in different jurisdictions require or accept *in vitro* mutagenicity assessments in cultured mammalian cells. For example, the United States Federal Insecticide, Fungicide, and Rodenticide Act (US FIFRA) Pesticide Assessment Guidelines require that *in vitro* mammalian cell mutagenicity tests are performed (Jaeger, 1984). Similarly, the Canadian Environmental Protection Act (CEPA) requires an *in vitro* test for gene mutation, with and without metabolic activation, for certain substances not on the Domestic Substances List (DSL) (Minister of Justice, 2018). The United States Food and Drug Administration (USFDA) Redbook, the European Regulation on Registration, Evaluation, Authorisation and Restriction of Chemicals (REACH), and the International Conference on Harmonization of Technical Requirements for Pharmaceuticals for Human Use (ICH) guidance on genotoxicity testing and data interpretation for pharmaceuticals intended for human use S2 (R1), and Japan's Chemical Substance Control Law all require, under certain conditions, an *in vitro* assessment of either chromosomal damage or mutagenesis (USFDA, 2007; METI, 2009; ICH, 2011; ECHA, 2017).

The mammalian cell genotoxicity assays that are currently used for regulatory assessments have all been extensively validated. Indeed, assays routinely used for regulatory evaluations and decision‐making, such as the *tk* gene mutation assay, have internationally accepted Organization for Economic Cooperation and Development (OECD) test guidelines (TG). However, there are only two OECD TGs for *in vitro* mutagenicity assessment in cultured mammalian cells, the aforementioned *tk* locus gene mutation test (i.e., TG 490), and the *Hprt*/*xprt* mutation test (i.e., TG 476; OECD, 2016a, 2016c). Although these assays have a long history of successful use for chemical safety assessments, they have several shortcomings, some of which are associated with the inherent traits of the cell lines used (e.g., L5178Y, TK6, Chinese hamster ovary, Chinese hamster lung, and Chinese Hamster V79 cell lines). These include lack of metabolic competence (OECD, 2016a, 2016c), aberrant karyotypes (Storer et al., 1997), and/or genomic instability (Lorge et al., 2016). Moreover, these assays require laborious, time‐consuming isolation and enumeration of mutant clones (OECD, 2016a, 2016c). To provide alternatives, immortalized cell lines derived from transgenic rodents such as the MutaMouse have been used to develop *in vitro* mammalian cell mutagenicity assays. One example is the *in vitro* mutagenicity assessment assay conducted in MutaMouse FE1 lung epithelial cells. This assay shows considerable promise for routine *in vitro* assessment of mutagenicity, and the assay is now partially validated (Maertens et al., 2017; Hanna, 2018). Although FE1 cells have some endogenous metabolic capacity (e.g., cytochrome P450 [CYP] 1A1), and can convert some genotoxic agents into reactive metabolites (e.g., benzo[*a*]pyrene [BaP]), the cells do not have a full complement of Phase I and II metabolic enzymes (White et al., 2003; Maertens et al., 2017; Hanna, 2018). In contrast, primary hepatocytes (PHs) from transgenic rodents, including the MutaMouse and the pUR288 *lacZ* Plasmid Mouse, have a more extensive complement of Phase I and II enzymes; as such, they are excellent candidates for the development of an *in vitro* mammalian cell mutagenicity assay [Chen et al., 2010; Zwart et al., 2012; Luijten et al., 2016].

Although *in vitro* mutagenicity tests based on cells (e.g., PHs) from transgenic rodents such as the MutaMouse and *lacZ* Plasmid Mouse show considerable promise, assays used for regulatory purposes must be validated to ensure adequate performance and reliability. The OECD TG program specifies criteria for the validation of novel toxicological test procedures. More specifically, to assess the performance and reliability of a novel test, the OECD requires the generation of information regarding test definition, intralaboratory variability, interlaboratory transferability, interlaboratory reproducibility, predictive capacity, applicability domain, and performance standards (OECD, 2005). In Part I of this two‐part series, we characterized MutaMouse PHs, demonstrating that they proliferate in culture, are karyotypically stable, carry the *lacZ* transgene vector for enumeration of chemically induced mutations, and express a comprehensive complement of Phase I and II metabolic enzymes (e.g., CYP 1A1, CYP 1A2, CYP 2B, CYP 2E1, CYP 3A, sulfotransferases (SULTs), UDP‐glucuronosyl transferases (UGTs), glutathione‐*S*‐transferases (GSTs), *N*‐acetyltransferases, *etc*.; Cox et al., 2018).

This study (i.e., Part II) aims to elucidate the predictive capacity and applicability domain of the assay. The predictive capacity is the ability of an assay to accurately predict the intended endpoint (e.g., gene mutation), whereas the applicability domain refers to the range of chemicals that can be reliably assessed (OECD, 2005). As MutaMouse PHs are metabolically most active in the first 24 h of culture, and this is followed by a period of proliferation (doubling time of 22.5 ± 3.3 h), the assay protocol includes a 6 h exposure to the chemical of interest within the first 24 h, followed by a sampling time of 72 h (Cox et al., 2018). The chemicals selected for the performance evaluation include known mutagens, known nonmutagens, and compounds that have been reported to elicit a misleading positive *in vitro* that is not manifested *in vivo* (Kirkland et al., 2008; Kirkland et al., 2016). These chemicals (i.e., *t*‐butylhydroquinone and eugenol), which are not DNA‐reactive, can indirectly elicit genotoxicity *in vitro*. The positive *in vitro* results are likely artifacts due to strain of cells used, cytotoxicity, or perturbations to the cell culture conditions (Fowler et al., 2012a, 2012b). Most of the chemicals investigated, which cover a range of chemical classes, have been suggested by the European Centre for the Validation of Alternative Methods (ECVAM) for the validation of novel *in vitro* genotoxicity assays (Kirkland et al., 2008, 2016). More specifically, in an effort to characterize and evaluate assay performance and applicability domain, we have examined a mutagenic nitrosourea, an acridine mutagen, a polycyclic aromatic hydrocarbon (PAH), an aromatic amine (AA), a heterocyclic amine (HA), a mycotoxin, two nitroarenes, and a nitrosamine, each of which has a unique mode of action and unique metabolic requirements. The test set of chemicals included four nonmutagens, two of which yield spurious positives in *in vitro* assays, as described above. Collectively, these assessments constitute an initial characterization of the performance and applicability domain (i.e., prevalidation) of the *in vitro* mutagenicity assay in MutaMouse PHs.

## MATERIALS AND METHODS

### Materials and Reagents

The CAS numbers and sources of all test chemicals are presented in Table 1. Dulbecco's modified Eagle's medium (DMEM), William's E medium, phosphate‐buffered saline (PBS), fetal bovine serum (FBS), epithelial growth factor (EGF), penicillin–streptomycin reagent, Hank's balanced salt solution (HBSS), proteinase K, trypan blue, and SYTOX® green were obtained from Life Technologies Inc. (Burlington, Ontario). Corning® Biocoat™ type I collagen‐coated culture dishes. CIzyme™ collagenase HA and BP protease were obtained from VitaCyte LLP (Indianapolis, Indiana). Dexamethasone, human insulin, dimethylsulphoxide (DMSO), Percoll®, bovine serum albumin (BSA), and IGEPAL CA‐630 were obtained from Sigma‐Aldrich Canada Co. (Oakville, Ontario). Phenyl‐β‐d‐galactopyranoside (P‐Gal) was obtained from MJS BioLynx (Brockville, Ontario). TransPak Packaging Extract was obtained from Agilent Technologies Canada (Mississauga, Ontario).

**Table 1:** Sources of Chemicals Used for *In Vitro* Exposures of MutaMouse PHs

Chemical	CAS number	Source
ENU	759‐73‐9	Sigma‐Aldrich (Oakville, Ontario)
ICR 191	17070‐45‐0	Sigma‐Aldrich (Oakville, Ontario)
BaP	50‐32‐8	Moltox (Boone, North Carolina)
AFB1	1162‐65‐8	Sigma‐Aldrich (Oakville, Ontario)
2‐AAF	53‐96‐3	Sigma‐Aldrich (Oakville, Ontario)
PhIP	105650‐23‐5	Moltox (Boone, NC)
1,8‐DNP	42397‐65‐9	Courtesy of Dr I. Lambert (Carleton University)
3‐NBA	17117‐34‐9	Courtesy of Dr V. Arlt (King's College London)
DMN	62‐75‐9	Sigma‐Aldrich (Oakville, Ontario)
Ampicillin trihydrate	7177‐48‐2	Sigma‐Aldrich (Oakville, Ontario)
D‐Mannitol	69‐65‐8	Sigma‐Aldrich (Oakville, Ontario)
TBHQ	1948‐33‐0	Sigma‐Aldrich (Oakville, Ontario)
Eugenol	97‐53‐0	Sigma‐Aldrich (Oakville, Ontario)

ENU, *N*‐ethyl‐*N*‐nitrosourea; 191, 6‐chloro‐9‐[3‐(2‐chloroethylamino)propylamino]‐2‐methoxyacridine dihydrochloride; BaP, benzo[a]pyrene; AFB1, aflatoxin B_1_; 2‐AAF, 2‐acetylaminofluorene; PhIP, 2‐amino‐1‐methyl‐6‐phenylimidazo[4,5‐b]pyridine; 1,8‐DNP, 1,8‐dinitropyrene; 3‐NBA, 3‐nitrobenzanthrone; DMN, dimethylnitrosamine; TBHQ, *tertiary* butylhydroquinone.

### Isolation and Culture of PHs

Female MutaMouse specimens were bred and maintained locally under conditions approved by the Health Canada Ottawa Animal Care Committee. Fresh MutaMouse PHs were isolated as specified in the companion manuscript (Cox et al., 2018). Briefly, cells were obtained using a two‐step collagenase technique with the addition of a Percoll® isodensity purification step (Seglen, 1976; Kreamer et al., 1986). The cells were plated at a density of 1.2 × 10^6^ cells/dish onto 100 mm collagen‐coated culture dishes using Attachment Medium (20 U/L human insulin, 4 × 10^−6^ mg/mL dexamethasone, 10% FBS, and 100 U/mL penicillin–streptomycin in DMEM), and incubated at 37°C and 5% CO_2_. Two hours (*t* = 2 h) following plating, the Attachment Medium was replaced with serum‐free medium (SFM; 10 mM HEPES, 2 mM l‐glutamine, 10 mM pyruvate, 0.35 mM l‐proline, 20 U/L human insulin, 4 × 10^−6^ mg/mL dexamethasone, 0.01 μg/mL EGF, and 100 U/mL penicillin–streptomycin in Williams Medium E), and the plates were incubated at 37°C and 5% CO_2_.

### Chemical Exposure and DNA Isolation

MutaMouse PHs were exposed to test chemicals as described by Chen et al. (2010), with some modifications. Briefly, stock solutions of the chemicals described in Table 1 were prepared in DMSO. After 18 h of culture, MutaMouse PHs were exposed to the chemicals of interest in SFM with 1% DMSO for 6 h at 37°C and 5% CO_2_. Three biological replicates (i.e., separate experiments using PHs from three different donor mice) were used for each test chemical. Following exposure, the medium was replaced with fresh SFM and the hepatocytes were incubated for a further 72 h prior to lysis and DNA isolation.

Following the 72 h sampling period, the SFM was replaced with lysis buffer (10 mM Tris pH 7.6, 10 mM ethylenediaminetetraacetic acid [EDTA], 150 mM sodium chloride, 1% sodium dodecyl sulphate [SDS], and 1 mg/mL proteinase K). The DNA was isolated by phenol chloroform extraction as previously described with an additional chloroform step (Gingerich et al., 2014). DNA was precipitated with ethanol, spooled onto a sealed Pasteur pipette, washed with 70% ethanol, dried, dissolved in TE^−4^ buffer (10 mM Tris pH 7.6 and 0.1 mM EDTA), and stored at 4°C.

### Mutant Frequency (MF) Determination

The frequency of *lacZ* mutants was determined using the P‐Gal positive selection method as previously described (Vijg & Douglas, 1996; Lambert et al., 2005; Chen et al., 2010; Gingerich et al., 2014). Briefly, TransPak was used to retrieve and package bacteriophage λgt10*lacZ* vectors from MutaMouse PH DNA. *E. coli* cells (*E. coli* C lacZ−, galE−, recA−, Kanr, pAA119; Gossen et al., 1992) were allowed to adsorb the phage particles; cells were plated with P‐Gal selective medium and incubated overnight at 37°C. Plaques were scored manually, and MF was calculated as the ratio of mutant plaque‐forming units (pfu) to total pfu determined from nonselective plates (i.e., without P‐Gal).

### Cytotoxicity Determination

Cytotoxicity was measured using the relative increase in nuclear counts (RINC) metric. RINC was quantified by flow cytometry as described previously with some modifications (Nüsse et al., 1994; Avlasevich et al., 2006; Bryce et al., 2007; Cox et al., 2018). Briefly, cultured hepatocytes were lysed using Lysis Buffer I (0.584 mg/mL NaCl, 1 mg/mL sodium citrate, 0.5 μL/mL IGEPAL, 0.7 U/mL RNase A, and 0.5 μM SYTOX® green nucleic acid stain). Following a 1 h incubation, Lysis Buffer II (85.6 mg/mL sucrose, 15 mg/mL citric acid, and 0.5 μM SYTOX® green nucleic acid stain) was added to the plates. To normalize nuclei counts, 150 μL of a suspension of 6 μm fluorescently labeled polystyrene microspheres was added to each sample of lysate. Ploidy was normalized as described previously (Cox et al., 2018). The microspheres have excitation/emission maxima of 488/515 nm (Cell Sorting Set‐up Beads for Blue Lasers, Life Technologies, Burlington, Ontario). Each microsphere‐lysate sample was diluted 1:10 prior to flow cytometric analysis. Data were acquired using a BD Biosciences FACScalibur flow cytometer (BD Biosciences, Mississauga, Ontario) equipped with a 488 nm laser. Instrumentation settings and data acquisition were facilitated using CellQuest Pro software (BD Biosciences). Data analysis was performed using Flowing Software version 2.5.1 (Turku Centre for Biotechnology, Turku, Finland). SYTOX® green and bead fluorescence emission were captured in the FL1 channel (530/30 band‐pass filter). Events were scored as nuclei following the application of key criteria (i.e., within a side scatter (SSC) vs forward scatter (FSC) region, within a region that excludes doublets, and within an FSC vs. FL1 region).

RINC values were calculated using a modification of the relative increase in cellular counts (RICC) formula (OECD, 2016b):

$$\text{RINC}=\frac{\text{Increase in relative number of nuclei in treated cultures}\;\left(\text{final}-\text{initial}\right)}{\text{Increase in relative number of nuclei in control cultures}\;\left(\text{final}-\text{initial}\right)},$$

wherein the initial count was obtained at the beginning of the exposure period and the final count was obtained 72 h following the end of the exposure period.

In accordance with the OECD test guidelines for the *in vitro* mammalian cell gene mutation assays using the *Hprt*, *Xprt*, and thymidine kinase genes, any positive responses elicited from concentrations with RINC values lower than 0.2 were interpreted with caution (OECD, 2016a, 2016c).

### Statistical Analyses

The *lacZ* MF data were analyzed in using RStudio version 1.0.136 (RStudio, Boston, MA) software using the glm function. The quasi‐Poisson distribution family was used to account for overdispersion, and the offset was designated as the natural log of total pfu (Haynes, 1989). Type 1, or sequential analysis, was employed to examine the statistical significance of the chemical treatment (i.e., Chi‐squared test), and custom contrasts statements were employed to evaluate the statistical significance of responses at selected doses or concentrations (Arlt et al., 2008). The resulting *P* values were corrected for multiple comparisons using the Bonferroni method. *P* values were considered to be significant if they were <0.05. Results for a given chemical were deemed positive if a significant response was obtained for the Chi‐squared test for overall treatment effect and at least one concentration yielded a significant MF increase above the concurrent vehicle control. A negative result was called if neither of these conditions were met. A chemical was deemed equivocal if only one of these conditions was met.

### Benchmark Dose (BMD) Modeling

BMD analysis was performed on all positive *lacZ* MF data using PROAST version 65.5 in R. The analysis employed chemical as a covariate. Both exponential and Hill nested model families were fit to the data. A benchmark response (BMR) of 100% (i.e., a twofold MF increase over control) was selected, as it has been previously used for the assessment of *lacZ* MF data; it lies within the range of observed results, thus allowing for optimal resolution of confidence intervals (Wills et al., 2016; Long et al., 2018).

## RESULTS

**Figure 1:**
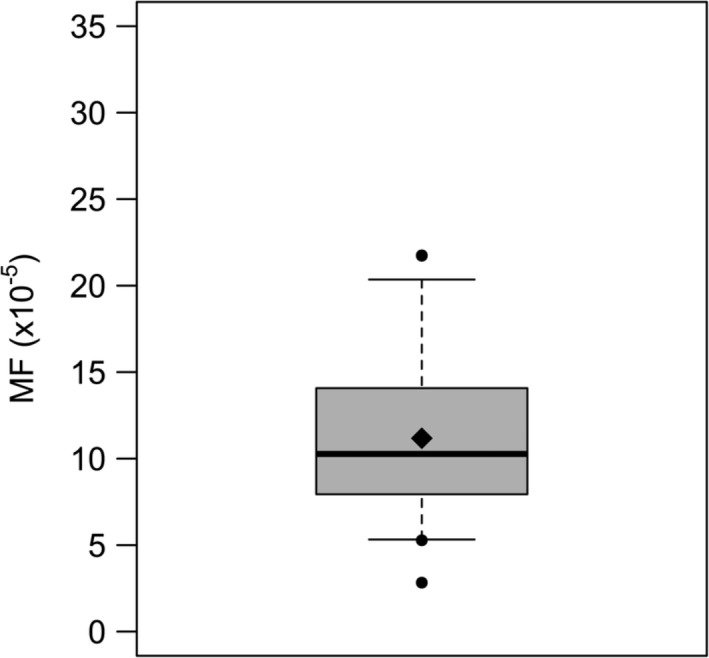
Spontaneous *lacZ* mutant frequency (MF) for MutaMouse primary hepatocytes (PHs). The solid line represents the median (10.7 × 10^−5^), the diamond represents the mean (11.0 × 10^−5^), the box limits represent the 25th and 75th percentiles (7.8 × 10^−5^ and 14.0 × 10^−5^, respectively), the whiskers represent the 5th and 95th percentiles (5.3 × 10^−5^ and 18.7 × 10^−5^, respectively), and the solid circles represent outliers that exist beyond the 5th and 95th percentiles. *N* = 32. Standard error of the mean (SEM) = 0.80 × 10^−5^.

The vehicle control data (Fig. 1) show a statistically normal distribution with a mean MF of 11.0 × 10^−5^ (SEM = 0.80 × 10^−5^, *N* = 32), and 5th and 95th percentiles of 5.3 × 10^−5^ and 18.7 × 10^−5^, respectively. These data were compiled from all available experiments (*N* = 32) performed over the course of four years.

**Figure 2:**
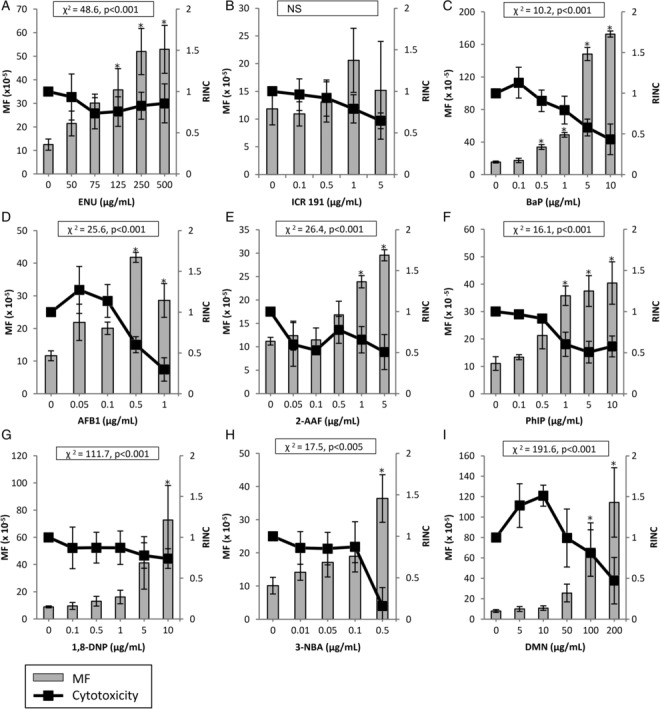
Induced *lacZ* transgene mutant frequency (MF) in MutaMouse primary hepatocytes (PHs) exposed to known mutagens. MutaMouse PHs were exposed to ENU (**A**), ICR 191 (**B**), BaP (**C**), AFB1 (**D**), 2‐AAF (**E**), PhIP (**F**), 1,8‐DNP (**G**), 3‐NBA (**H**), and DMN (**I**). Gray bars represent MF ± standard error of the mean (SEM) and black squares represent relative increases in nuclear counts (RINC) ± SEM, a measure of cytotoxicity. Asterisks indicate MF values that are significantly elevated relative to control (*P* < 0.01). Inset boxes show statistical results for the overall concentration–response relationship. *N* = 3 for all observations, except for 0.05 μg/mL AFB1, wherein *N* = 2 for MF data. NS, not significant.

The sensitivity of MutaMouse PHs in an *in vitro* gene mutation assay was investigated following exposure to nine well‐characterized mutagens. For all chemicals tested, the MF values of treated cells were compared to concurrently run vehicle controls to determine significance. Of the mutagens assessed, all but ICR 191 yielded a significant MF increase over control for at least one concentration, and, more importantly, a significant overall treatment effect (Fig. 2B). The three top concentrations of ENU elicited significant MF increases over control with a maximum response of 4.2‐fold at 1,000 μg/mL (Fig. 2A). A significant MF increase was observed in MutaMouse PHs exposed to BaP at the four highest concentrations tested, culminating in a maximum fold‐increase of ~11‐fold at 10 μg/mL (Fig. 2C). AFB1 yielded a significant MF increase at 0.5 μg/mL with a 3.6‐fold MF increase, which falls to a significant 2.5‐fold MF increase at 1 μg/mL (Fig. 2D). This trend is accompanied by increased cytotoxicity as the RINC falls from 0.6 to 0.3. 2‐AAF yielded a significant response at the top two concentrations tested with a 2.6‐fold MF increase above control at 5 μg/mL (Fig. 2E). PhIP yielded significant MF increases at the top three concentrations tested with a 3.7‐fold increase at 10 μg/mL (Fig. 2F). 1,8‐DNP yielded a significant MF increase of 3.6‐fold at 10 μg/mL (Fig. 2G). 3‐NBA elicited a significant MF fold‐increase of 8.2 at 0.5 μg/mL, and this was accompanied by a sharp increase in cytotoxicity (Fig. 2H). DMN showed significant increases in MF at the top two concentrations tested, with a maximum fold‐increase of 14.4‐fold at 200 μg/mL (Fig. 2I).

**Figure 3:**
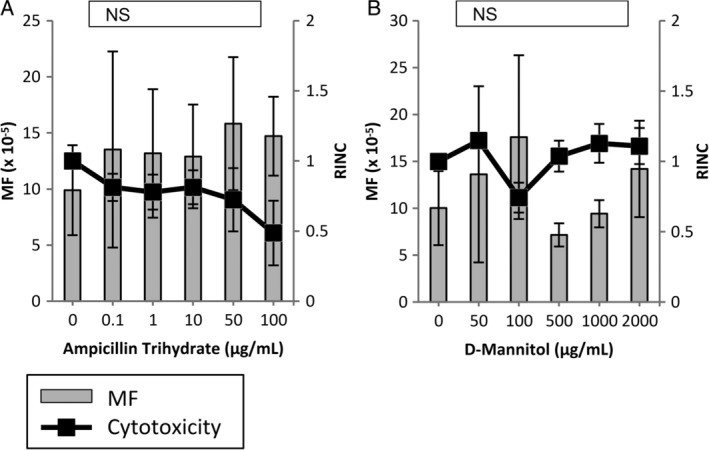
Induced *lacZ* transgene mutant frequency (MF) in MutaMouse primary hepatocytes (PHs) exposed to non‐DNA‐reactive chemicals (i.e., known nonmutagens). MutaMouse PHs were exposed to ampicillin trihydrate (**A**) and d‐mannitol (**B**). Gray bars represent MF ± standard error of the mean (SEM) and black squares represent relative increases in nuclear counts (RINC) ± SEM, a measure of cytotoxicity. Inset boxes show statistical results for the overall concentration–response relationship. *N* = 3 for all observations. NS, not significant.

**Figure 4:**
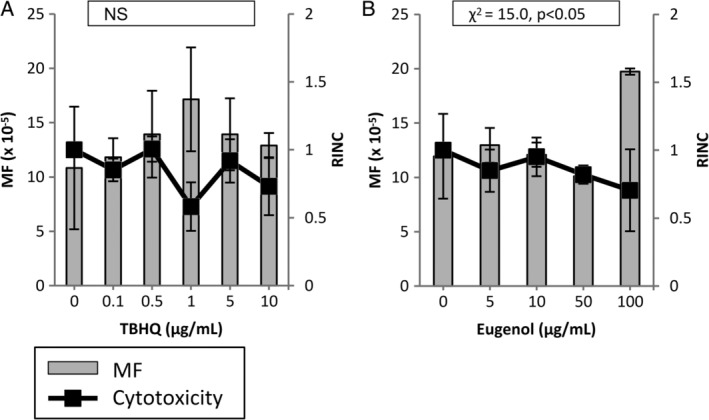
Induced *lacZ* transgene mutant frequency (MF) in MutaMouse primary hepatocytes (PHs) exposed to non‐DNA‐reactive chemicals that have been shown to elicit positive results in other *in vitro* genotoxicity assays (i.e., misleading positives). MutaMouse PHs were exposed to TBHQ (**A**) and Eugenol (**B**). Gray bars represent MF ± standard error of the mean (SEM) and black squares represent relative increases in nuclear counts (RINC) ± SEM, a measure of cytotoxicity. Inset boxes show statistical results for the overall concentration–response relationship. *N* = 3 for all observations. NS, not significant.

The MutaMouse PH gene mutation assay did not yield any significant MF increases for any of the nonmutagenic chemicals tested, including the aforementioned misleading positives (Figs. 3 and 4). The results obtained for eugenol demonstrate a significant treatment effect, despite the absence of a significant response at any concentration tested (Fig. 4B). Eugenol could not be tested at higher concentrations due to cytotoxicity.

**Figure 5:**
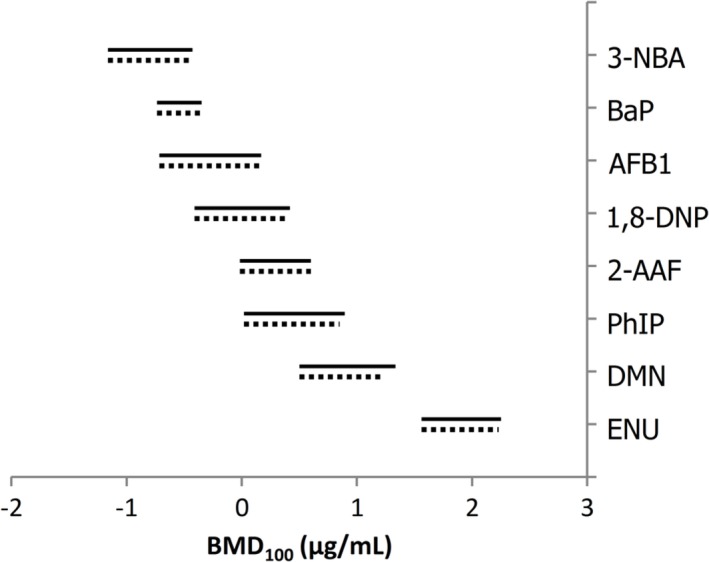
Benchmark dose (BMD) values (i.e., BMD_100_) with two‐sided 90% confidence intervals generated using BMD covariate analysis of MutaMouse primary hepatocyte (PH) mutant frequency (MF) dose–response data. BMD analysis was only conducted for agents shown to elicit significant positives responses. Solid lines represent the fitted exponential model, whereas dotted lines represent the Hill model.

To rank the potencies of all chemicals that elicited a positive response in the MutaMouse PH gene mutation assay, the confidence intervals of the BMD_100_ values were plotted in order of decreasing potency (i.e., from lowest to highest BMD_100_; Fig. 5). There was little to no difference between the exponential and Hill models. The ranking of chemicals from most to least potent was: 3‐NBA, BaP, AFB1, 1,8‐DNP, 2‐AAF, PhIP, DMN, followed by ENU. The BMD_100_, BMDL, and BMDU values for each chemical are presented in Supporting Information Table SI.

## DISCUSSION

This study aimed for an initial characterization of the performance and applicability domain of the *in vitro* gene mutation assay in MutaMouse PHs. Along with Part I, which extensively characterized cultured MutaMouse PHs, this study represents the first step toward the validation and acceptance of this test method. In this preliminary validation study, initial conclusions regarding the predictive capacity and applicability domain were obtained by challenging the assay with a set of mutagenic and nonmutagenic chemicals.

The OECD assay validation guidelines require the careful examination of historical negative controls, thus throughout the development of the MutaMouse PH assay, all spontaneous MF values have been recorded and compiled. A total of 32 spontaneous MF values were compiled from separate experiments performed over 4 years (Fig. 1). The mean spontaneous MF value observed for MutaMouse PHs (i.e., 11.0 × 10^−5^ ± 0.80 × 10^−5^) was comparable to that observed for PHs from the pUR288 *lacZ* plasmid mouse, which has a mean spontaneous MF of 9.1 × 10^−5^, and a 95th percentile of 12.0 × 10^−5^, (Luijten et al., 2016). The spontaneous MF observed for MutaMouse PHs is also similar to what is observed in MutaMouse liver *in vivo* (i.e., 6.31 × 10^−5^ ± 1.3 × 10^−5^) (Lemieux et al., 2011). Previous work in MutaMouse PHs, using a different isolation and culture protocol, elicited a mean spontaneous MF value of 14.2 × 10^−5^ ± 1.7 × 10^−5^ across 11 biological replicates (Chen et al., 2010). The FE1 cell line, derived from MutaMouse lung epithelium has a higher mean spontaneous MF of 51 ± 0.9 × 10^−5^ (*N* = 460; Maertens et al., 2017). With a mean spontaneous MF value of 11.0, a 95th percentile of 18.7, and a conventional *α* level of 0.05 for the *P* value, the MutaMouse PH gene mutation assay can reliably detect significant increases in MF that are ~1.7‐fold higher than the vehicle control (Hayashi et al., 2011).

In Part I, it was shown that cultured MutaMouse PHs encompass a virtually full complement of Phase I and Phase II metabolic enzymes. To assess the functional utility of the PHs' complement of metabolic enzymes, in comparison to conventional gene mutation assays, this study examined chemicals with a range of metabolic activation pathways and modes of action (Supporting Information Table SII). Table 2 compares the responses of the chemicals assessed in this study to their responses in the pUR288 *lacZ* plasmid mouse PH gene mutation assay, the *in vitro* MutaMouse FE1 gene mutation assay, the MLA, and the HPRT test. To our knowledge, no gene mutation assay using an immortalized or transformed cell line can cover this range of chemicals without the addition of an exogenous source of mammalian metabolic enzymes (i.e., rodent liver S9).

**Table 2:** Comparisons of Chemical Responses Across a Range of *In Vitro* Gene Mutation Assays. Citations for the Indicated Responses are Provided in the Text

Chemical	MutaMouse PHs	pUR288 *lacZ* Plasmid Mouse PHs	MutaMouse FE1	MLA	HPRT
*True positive*					
ENU	Positive	Positive	Positive (−S9)	Positive (−S9)	Positive (−S9)
ICR 191	Negative	NT	Positive (−S9)	Positive (−S9)	Positive (−S9)
BaP	Positive	Positive	Positive (±S9)	Positive (+S9)	Positive (+S9)
AFB1	Positive	NT^a^	Positive (±S9)	Positive (+S9)	Positive (+S9)
2‐AAF	Positive	NT	Positive (±S9)	Positive (±S9)	Positive (+S9)
PhIP	Positive	NT	Positive (+S9)	Positive (+S9)^b^	Positive (+S9)
1,8‐DNP	Positive	NT	NT	Positive (−S9)	Pos/Neg^c^ (±S9)
3‐NBA	Positive	NT	Positive (±S9)	Positive^d^	Positive^d^
DMN	Positive	NT	Negative (+S9)	Positive (+S9)	Positive (+S9)
*True negative*					
Ampicillin trihydrate	Negative	NT	Negative (−S9)	Negative (±S9)	NT
D‐Mannitol	Negative	Negative	Negative (−S9)	Negative (±S9)	NT
*Misleading positive*					
TBHQ	Negative	NT	Negative (±S9)	NT	NT
Eugenol	Negative	NT	Negative (±S9)	Positive (−S9)	NT

a NT, not tested.

b Human TK6 assay.

c Both positive and negative responses have been observed.

d Mutagenicity was assessed at *tk* and *hprt* loci in the human lymphoblast cell lines, h1A1v2 and MCL‐5, that have been transfected with CYP1A1, and CYP1A2, CYP2A6, CYP2E1, and CYP3A4, respectively

As a nitrosourea and a strong alkylating agent, ENU is a direct‐acting agent and, thus, does not require metabolic activation to become DNA‐reactive (Doak et al., 2007). It is known to cause transitions and transversions following *O* ^2^‐ and *O* ^4^‐alkylation of thymine residues, and *O* ^6^‐alkylation of guanine residues, respectively (Douglas et al., 1995). It yields a positive response in conventional gene mutation assays, such as the MLA and HPRT test, without the addition of induced rodent S9 (Nishi et al., 1984; Chen et al., 2002; Doak et al., 2007; Kirkland et al., 2008; Kirkland et al., 2016). In this study, ENU induced a concentration‐dependent increase in MF culminating in a 4.2‐fold increase over control (Fig. 2A). The magnitude of the observed response is comparable to what is seen in pUR288 *lacZ* plasmid mouse PHs and MutaMouse FE1 cells (i.e., ~4‐ and ~6‐fold increases, respectively) (White et al., 2003; Luijten et al., 2016).

ICR 191 is an acridine half‐mustard that, like ENU, interacts with DNA without metabolic conversion to a DNA‐reactive metabolite (Ferguson & Denny, 1990). Unlike ENU, ICR 191 is known to intercalate within the DNA molecule and cause +1 frameshifts in regions with consecutive guanine residues (Taft et al., 1994). Like ENU, ICR 191 is positive in conventional gene mutation assays, and the MutaMouse FE1 gene mutation assay, without the addition of S9 (Nishi et al., 1984; Doerr et al., 1989; Mitchell et al., 1997; White et al., 2003). Interestingly, ICR 191 did not yield a significant increase in MF in MutaMouse PHs, nor was a significant treatment effect detected (Fig. 2B). It should be noted that the variability at the two highest concentrations was relatively high compared to the other chemicals tested. Uninduced murine liver S9 has been shown to dramatically reduce the mutagenicity of ICR 191 in the Ames test (i.e., Salmonella strain TA1537), whereas untreated murine lung S9 had little effect on mutagenicity (De Flora et al., 1982). This suggests that mouse liver metabolic enzymes are better able to detoxify ICR 191 than mouse lung metabolic enzymes, which may account for the discrepancy between the MutaMouse PH response and the result observed for Mouse FE1 pulmonary cells (White et al., 2003) (Table 2). The high metabolic competency of MutaMouse PHs may be reducing the mutagenicity of ICR 191 in this *in vitro* system.

BaP is one of the several highly mutagenic polycyclic aromatic hydrocarbons (PAH) that are by‐products of incomplete combustion processes. BaP is considered a prototypical PAH that is known to form DNA adducts following Phase I metabolic activation involving CYPs 1A1, 1A2, and 3A, as well as epoxide hydrolase (Jeffrey, 1985; Bauer et al., 1995; Kim et al., 1998). These enzymatic reactions convert BaP to a DNA‐reactive metabolite, such as BaP‐diol‐epoxide (BPDE), to form adducts with guanine and adenine residues (Jeffrey, 1985). BaP is typically a strong positive in the MLA and HPRT tests with the addition of S9 (Bradley et al., 1981; Oberly et al., 1990; Mitchell et al., 1997; Kirkland et al., 2008; Kirkland et al., 2016). BaP induced a 11‐fold concentration‐dependent MF increase over control in MutaMouse PHs, which is comparable to what is observed in pUR288 *lacZ* plasmid mouse PHs (i.e., 9.8‐fold), but less than what is observed in MutaMouse FE1 cells (i.e., ~25‐fold) (White et al., 2003; Zwart et al., 2012) (Fig. 2C).

AFB1 is a mutagenic mycotoxin, food contaminant, and known human carcinogen that is metabolized to AFB1‐8,9‐epoxide, a DNA‐reactive metabolite, that is capable of forming adducts with guanine residues (Essigmann et al., 1977; Gallagher et al., 1994). This activation is carried out by Phase I CYPs that are present in MutaMouse PH cultures, in particular, CYPs 1A2 and 3A. Like BaP, AFB1 is generally a strong positive in the MLA and HPRT assay with S9 (Bradley et al., 1981; Preisler et al., 2000; Kirkland et al., 2008; Kirkland et al., 2016). AFB1 induced a 3.6‐fold concentration‐dependent increase over control in MutaMouse PHs, which is similar to the ~three‐fold increase seen in MutaMouse FE1 cells (Hanna, 2018) (Fig. 2D).

2‐AAF and PhIP are both mutagenic aromatic amines that undergo similar metabolic activation pathways to become DNA‐reactive. 2‐AAF is a prototypical aromatic amine in mutagenesis studies (Heflich & Neft, 1994). The heterocyclic amine, PhIP, is a by‐product of the Maillard, or browning, reaction that occurs during the cooking of meat (Jägerstad et al., 1983). Aromatic amines, such as 2‐AAF and PhIP, require both Phase I enzymes, such as CYP 1A1 and 1A2, as well as Phase II enzymes, such as, SULT, NAT, and/or UGT to generate DNA‐reactive nitrenium or carbenium ions (Heflich & Neft, 1994; Schut & Snyderwine, 1999; Cai et al., 2016). Both 2‐AAF and PhIP are positive in the MLA and HPRT tests with the addition of S9 (Oberly et al., 1990; Morgenthaler & Holzhäuser, 1995; Mitchell et al., 1997; Kirkland et al., 2008; Kirkland et al., 2016). 2‐AAF and PhIP induced 2.6‐ and 3.7‐fold increases, respectively, in MF above control in MutaMouse PHs (Fig. 2E,F). Once again, these values are comparable to the ~1.7‐ and ~5.5‐fold increases observed in MutaMouse FE1 cells following exposure to 2‐AAF and PhIP, respectively (White et al., 2003; Hanna, 2018).

Nitroarenes, such as 1,8‐DNP and 3‐NBA, are mutagenic compounds found in both diesel and gasoline engine exhaust (IARC, 2014). The metabolic activation pathway of 1,8‐DNP is presumed to require both nitroreduction and acetyltransferase metabolic activity to yield the DNA‐reactive metabolite, *N*‐acetoxy‐1‐amino‐8‐nitropyrene, that is capable of forming guanine adducts (IARC, 2014). Similarly, the proposed metabolic activation pathway for 3‐NBA involves nitroreductase activity, as well as *N‐*acetyltransferase and/or sulfotransferase catalytic activity to yield a highly reactive nitrenium ion that can form guanine or adenine adducts (Arlt et al., 2003; Arlt et al., 2005; IARC, 2014). This pathway may also involve CYP 1A1, 2A6, 2B6, or 3A4 oxidation, following reduction to 3‐aminobenzanthrone. The nitroreduction required for the activation of nitroarenes sets these chemicals apart from the other compounds examined in this study. Interestingly, in the companion manuscript, NAD(P)H dehydrogenase, quinone 1 (NQO1), a cytosolic enzyme capable of nitroreduction, was shown to be expressed in cultured MutaMouse PHs. 1,8‐DNP is positive in the MLA without the addition of S9, but its response in the HPRT test depends on the cell line in which it is tested (Edgar, 1985). When tested in the HPRT assay in CHO cells, it elicited a strong mutagenic response; however, in HepG2 cells, 1,8‐DNP yielded a negative response (Edgar & Brooker, 1985; Eddy et al., 1986). 3‐NBA has yielded positive results at *tk* and *hprt* loci in the human lymphoblast cell lines, MCL‐5 and h1A1v2, which have been transfected with human CYPs (Phousongphouang et al., 2000; Arlt et al., 2008). Both 1,8‐DNP and 3‐NBA yielded positive results in the MutaMouse PH gene mutation assay, with induced fold‐changes in MF of 8.2 and 3.6, respectively (Fig. 2G,H). Of the two, only 3‐NBA has been assessed in MutaMouse FE1 cells, yielding a ~five‐fold increase in MF above solvent control (Arlt et al., 2008).

Mutagenic nitrosamines, like DMN, are by‐products of some industrial processes and water treatment. They are also found in cigarette smoke and in some foods, such as cured meat and beer. DMN is activated by Phase I enzymes, including CYP 2E1, to methyldiazohydroxide, followed by potential formation of a diazonium ion that methylates nucleic acids, particularly guanine residues (Hoffmann & Hecht, 1985; Yamazaki et al., 1992; Chowdhury et al., 2012). DMN has yielded positive results in both the MLA and HPRT assays with the addition of S9 (Bradley et al., 1981; Mitchell et al., 1997; Kirkland et al., 2008; Kirkland et al., 2016). DMN induced a 14.4‐fold increase in MF above control in MutaMouse PHs (Fig. 2I), but, interestingly, did not elicit a significant positive response in MutaMouse FE1 cells in the presence or absence of S9 (Hanna, 2018).

This study included four nonmutagenic chemicals to offer some insight into the specificity of the MutaMouse PH gene mutation assay. d‐Mannitol and ampicillin trihydrate are known to be nonmutagenic, and are consistently negative in gene mutation assays (Mitchell et al., 1997; Kirkland et al., 2008; Kirkland et al., 2016; Maertens et al., 2017). These chemicals both yielded negative results in the MutaMouse PH gene mutation assay (Fig. 3A,B). TBHQ, one of the misleading positive chemicals assessed in this study, has not been tested in either of the more conventional gene mutation tests (i.e., MLA or HPRT), but has yielded a negative result in the FE1 assay (Maertens et al., 2017). Eugenol, another misleading positive chemical has elicited a positive result in the MLA test, but a negative result in the FE1 assay (Mitchell et al., 1997; Maertens et al., 2017). Both TBHQ and eugenol have tested positive in the *in vitro* chromosome aberration test and positive for micronucleus in p53‐deficient hamster cells. They have both also resulted in negative results for the *in vitro* micronucleus assay in p53‐functional human cells (NTP, 1982; NTP, 1986; Kirkland et al., 2008; Fowler et al., 2012a; Kirkland et al., 2016). TBHQ and eugenol are part of a larger subset of chemicals that are not DNA‐reactive, but are thought to disturb cell culture conditions or exert toxicity on cells *in vitro* in a way that leads to an apparent genotoxic response. These chemicals have both elicited negative results *in vivo*; however, these data are rather limited and mainly restricted to hematopoietic tissues (e.g., the bone marrow and blood) (Kirkland et al., 2016). Eugenol has also elicited negative results in the liver as measured by the unscheduled DNA synthesis (UDS) assay and the *in vivo* MutaMouse assay (Rompelberg et al., 1996a; Rompelberg et al., 1996b). Oxidative stress and cytotoxicity are thought to be the major factors influencing the positive results seen for TBHQ and eugenol, respectively (Kirkland et al., 2008; Fowler et al., 2012a; Fowler et al., 2012b; Kirkland et al., 2016). Neither TBHQ nor eugenol yielded a significant increase in MF over the solvent control in MutaMouse PHs at any concentration tested (Fig. 4A,B). Due to the relatively high RINC obtained at the top concentration of TBHQ, this chemical should be tested at a higher range of concentrations. Eugenol did elicit a significant result for the overall treatment effect (i.e., Chi‐squared for overall effect); however, this compound was too cytotoxic to test at higher concentrations (i.e., no DNA could be isolated for MF testing) and the effect appeared to be driven solely by the highest concentration tested. Following the criteria outlined in the Materials and Methods, ampicillin trihydrate, d‐mannitol, and TBHQ are negative and eugenol is equivocal in the MutaMouse PH gene mutation assay.

This study employed the BMD approach to quantitatively examine the MutaMouse PH gene mutation assay results and compare the responses of the various chemicals (Fig. 5). By fitting a model to concentration–response data, BMD analysis yields a concentration that elicits a specified response. The BMD covariate approach builds on this concept by incorporating a covariate, such as compound, to rigorously compare potencies within an endpoint (Wills et al., 2016). The use of a covariate refines BMD confidence intervals and improves the precision of BMD values (Slob & Setzer, 2014; Wills et al., 2016). The results revealed that slope, maximal response, and variance were conserved across all chemicals for both the fitted exponential and Hill models. When comparing the results obtained, no significant distinction in potency can be made between chemicals with overlapping confidence intervals. 3‐NBA, BaP, AFB1, and 1,8‐DNP appear to be the most potent chemicals in this assay, and it appears that there could be a link between potency and mode of action. As discussed above, 3‐NBA, BaP, AFB1, and 1,8‐DNP all undergo Phase I metabolic activation, and elicit similar forms of DNA damage (i.e., guanine and adenine adducts). 2‐AAF, PhIP, and DMN are less potent than 3‐NBA and BaP. 2‐AAF and PhIP are both aromatic amines with similar modes of activation involving both Phase I and Phase II metabolism, and genotoxic effects, as described above. DMN has a separate mode of action as it generally exerts its genotoxicity by methylating guanine residues following CYP 2E1 metabolism. ENU yielded the lowest BMD_100_ (i.e., potency), and, like DMN, it is known to be a DNA alkylating agent, albeit a direct‐acting one. Although this preliminary study only examines a small subset of mutagenic chemicals, the BMD covariate results indicate that DNA adduct‐forming promutagens requiring Phase I metabolic activation are the most potent in this assay, followed by DNA adduct‐forming promutagens requiring both Phase I and Phase II metabolic activation, and finally, DNA alkylating agents. The lower potency of ENU, relative to compounds forming bulky adducts, such as BaP, was also observed in the MutaMouse FE1 and pUR288 *lacZ* plasmid mouse PH *in vitro* gene mutation assays; however, ENU is more potent than BaP in the MutaMouse gene mutation assay in the liver and bone marrow *in vivo*, and of equivalent potency in the small intestine (Gocke et al., 2009; Luijten et al., 2016; Hanna, 2018; Long et al., 2018). The BMD covariate analysis presented herein provides further insight into the genotoxic effects of the compounds studied in metabolically competent cells; it also demonstrates the utility of the BMD covariate approach for comparative analysis of *in vitro* dose–response data.

It is important to note that the ability to employ *in vitro* tools to effectively and efficiently assess genotoxicity is a critical component of the evolving paradigm for “toxicity testing in the 21st century” (Krewski et al., 2010). This paradigm calls for adoption of high(er)‐throughput screening tools for efficient (geno)toxicity assessment and MOA determination. Moreover, effective adoption of novel *in vitro* tools is consistent with global initiatives aimed at replacing, reducing, and refining the use of animals for (geno)toxicity assessment and attendant regulatory decision‐making (European Commission, 2009; Adler et al., 2011). In this regard, *in vitro* mutagenicity assessment using MutaMouse PHs is aligned with global initiatives to modernize mutagenicity assessment. The *in vivo* MutaMouse assay can require more than 20 animals per compound, PHs isolated from a single MutaMouse specimen provide enough cells to test one to three chemicals. Nevertheless, it must also be noted that the MutaMouse PH mutagenicity assay described herein is nowhere near as efficient as some recently developed, high‐throughput *in vitro* genotoxicity reporter assays. These assays, such as the ToxTracker® assay (Hendriks et al., 2012), the MultiFlow® assay (Bryce et al., 2016), and the TGx‐DDI toxicogenomic biomarker assay (Buick et al., 2015), can rapidly and simultaneously assess multiple cellular responses indicative of DNA damage, thereby efficiently identifying genotoxicants and elucidating MOA. While such assays can indeed be categorized as high throughput, they cannot detect the endpoints that are requisite in the aforementioned legislative frameworks (i.e., CEPA, FIFRA, CSCL, etc.), i.e., mutations and/or chromosome damage. Thus, in the short to medium term, it will be necessary to develop and adopt high(er)‐throughput mutagenicity assays such as that presented herein.

This study, alongside Part I, constitutes an important first step toward the validation of the MutaMouse PH *in vitro* gene mutation assay. The positive responses elicited by BaP, AFB1, 2‐AAF, PhIP, DMN, 1,8‐DNP, and 3‐NBA illustrate that the MutaMouse PHs are capable of converting these mutagenic compounds to their reactive metabolites without the addition of exogenous S9. The results thus far indicate that the applicability domain of the assay encompasses chemicals that require Phase I and/or Phase II metabolism. This is consistent with the companion paper (i.e., Part I) where we demonstrated that MutaMouse PHs maintain maximal metabolic enzyme activity for at least the first 24 h in culture. As the exposures in this study take place from 18 to 24 h postisolation, we expected that the cells would be capable of activating promutagens during this time; indeed, that is what was observed.

Due to their karyotypically normal phenotype, metabolic capacity, and DNA‐repair proficiency, it is not unreasonable to assert that some direct‐acting chemicals may not be detected in this assay. The negative result for ICR 191 illustrates a gap in this assay's applicability domain. Testing of a larger set of both positive and negative chemicals, including more *in vitro* misleading positive chemicals and *in vitro* false‐positive chemicals (e.g., chemicals that are positive in the Ames test and negative *in vivo*) will further resolve the sensitivity (i.e., ability to correctly identify mutagens) and specificity (i.e., ability to correctly identify nonmutagens) of the assay, and extend the elucidation of the applicability domain. Although positive controls were not included in this preliminary study, it is recommended that BaP and PhIP be included in future studies as positive controls for Phase I and Phase II assessment, respectively, at 10 μg/mL. Future work includes the development of a cryopreservation protocol, as has been developed for pUR288 *lacZ* Plasmid Mouse PHs, to facilitate the use of these cells for routine screening; moreover, their distribution to laboratories interested in adopting the assay (Luijten et al., 2016). This study indicates that this system shows great promise as a metabolically competent complement to bacterial mutagenicity tests, particularly for compounds that require metabolic activation.

## STATEMENT OF AUTHOR CONTRIBUTIONS

Dr White and Ms Cox designed the study. Ms Cox conducted the laboratory experiments, for which Drs Zwart and Luijten provided essential input. Dr White and Ms Cox analyzed the data. Ms Cox prepared tables and draft figures. Ms Cox prepared the draft manuscript with important intellectual input from Drs White, Luijten, and Zwart. All authors approved the final manuscript.

## Supporting information


**Table SI** Summary of the BMD_100_ values, including 90% confidence intervals (i.e., BMDL and BMDU values) for all positive *lacZ* mutant frequency (MF) data using both the exponential and Hill models from the MutaMouse primary hepatocyte (PH) assay.
Table SII**.** Summary of the enzymes required for metabolic activation of the chemicals tested and the presence of these enzymes in MutaMouse primary hepatocytes (PHs) *in vitro*.
