# Supplementary material for: The development and prevalidation of an in vitro mutagenicity assay based on MutaMouse primary hepatocytes, Part II: Assay performance for the identification of mutagenic chemicals
Source: Environ Mol Mutagen. 2019 Feb 25;60(4):348–60. doi: 10.1002/em.22277 (PMC6593967; doi:10.1002/em.22277)
Supplement: Supplementary file 1 — Table SI Summary of the BMD100 values, including 90% confidence intervals (i.e., BMDL and BMDU values) for all positive lacZ mutant frequency (MF) data using both the exponential and Hill models from the MutaMouse primary hepatocyte (PH) assay. Table SII. Summary of the enzymes required for metabolic activation of the chemicals tested and the presence of these enzymes in MutaMouse primary hepatocytes (PHs) in vitro. [file EM-60-348-s001.docx]

Supplementary Tables:

**Supplementary Table I.** Summary of the BMD_100_ values, including 90% confidence intervals (i.e., BMDL and BMDU values) for all positive *lacZ* mutant frequency (MF) data using both the exponential and Hill models from the MutaMouse primary hepatocyte (PH) assay

| Chemical | BMD_100_ (µg/mL) | | BMDL (µg/mL) | | BMDU (µg/mL) | |
| --- | --- | --- | --- | --- | --- | --- |
|  | Exponential | Hill | Exponential | Hill | Exponential | Hill |
| 3-NBA | 0.15 | 0.15 | 0.0689 | 0.0689 | 0.373 | 0.363 |
| BaP | 0.3 | 0.3 | 0.184 | 0.184 | 0.446 | 0.446 |
| AFL | 0.47 | 0.47 | 0.193 | 0.193 | 1.47 | 1.47 |
| 1,8-DNP | 0.97 | 0.97 | 0.39 | 0.39 | 2.62 | 2.44 |
| 2-AAF | 1.9 | 1.9 | 0.964 | 0.964 | 3.96 | 3.96 |
| PhIP | 2.6 | 2.6 | 1.05 | 1.05 | 7.8 | 7.05 |
| DMN | 7.6 | 7.6 | 3.18 | 3.18 | 21.5 | 16.8 |
| ENU | 76 | 76 | 36.6 | 36.6 | 178 | 170 |

**Supplementary Table II.** Summary of the enzymes required for metabolic activation of the chemicals tested and the presence of these enzymes in MutaMouse primary hepatocytes (PHs) *in vitro*.

| Chemical | Enzymes required | Presence in MutaMouse PHs^a^ | | Reference |
| --- | --- | --- | --- | --- |
|  |  | Activity^b^ | Expression^c^ |  |
| BaP | CYP^d^ 1A1  CYP 1A2  CYP 3A  Epoxide hydrolase | Yes  Yes  Yes  ND | Yes  Yes  Yes  Yes | [Jeffrey, 1985; Bauer et al., 1995; Kim et al., 1998] |
| AFB1 | CYP 1A2  CYP 3A | Yes  Yes | Yes  Yes | [Gallagher et al., 1984] |
| 2-AAF and PhIP | CYP 1A1  CYP 1A2  SULT^e^  NAT^f^  UGT^g^ | Yes  Yes  Yes  ND  Yes | Yes  Yes  Yes  Yes  ND | [Schut and Snyderwine, 1999; Heflich and Neft, 1994; Cai et al., 2016] |
| 1,8-DNP | NQO1^h^  NAT | ND  ND | Yes  Yes | [IARC, 2014] |
| 3-NBA | NQO1  NAT  SULT | ND  ND  Yes | Yes  Yes  Yes | [IARC, 2014; Arlt et al., 2003; Arlt et al., 2005] |
| DMN | CYP 2E1 | ND | Yes | [Chowdhury et al., 2012; Yamazaki et al., 1992; Hoffmann and Hecht, 1985] |

^a^ Data from [Cox et al., 2018]

^b^ Activity measured by EROD for CYP 1A1/1A2 and LC-MS/MS for CYP 3A, SULT and UGT

^c^ Gene expression measured by RT-qPCR

^d^ CYP, cytochrome P450

^e^ SULT, sulfotransferase

^f^ NAT, *N*-acetyltransferase

^g^ UGT, UDP-glucuronosyltransferase

^h^ NQO1, NA(D)PH dehydrogenase, quinone 1
